# The Role of Lipolysis Stimulated Lipoprotein Receptor in Breast Cancer and Directing Breast Cancer Cell Behavior

**DOI:** 10.1371/journal.pone.0091747

**Published:** 2014-03-17

**Authors:** Denise K. Reaves, Katerina D. Fagan-Solis, Karen Dunphy, Shannon D. Oliver, David W. Scott, Jodie M. Fleming

**Affiliations:** 1 Department of Biology, North Carolina Central University, Durham, North Carolina, United States of America; 2 Department of Veterinary and Animal Sciences, University of Massachusetts, Amherst, Massachusetts, United States of America; 3 Department of Cell Physiology and Cell Biology, University of North Carolina at Chapel Hill, Chapel Hill, North Carolina, United States of America; University of North Carolina at Chapel Hill, United States of America

## Abstract

The claudin-low molecular subtype of breast cancer is of particular interest for clinically the majority of these tumors are poor prognosis, triple negative, invasive ductal carcinomas. Claudin-low tumors are characterized by cancer stem cell-like features and low expression of cell junction and adhesion proteins. Herein, we sought to define the role of lipolysis stimulated lipoprotein receptor (LSR) in breast cancer and cancer cell behavior as LSR was recently correlated with tumor-initiating features. We show that LSR was expressed in epithelium, endothelium, and stromal cells within the healthy breast tissue, as well as in tumor epithelium. In primary breast tumor bioposies, LSR expression was significantly correlated with invasive ductal carcinomas compared to invasive lobular carcinomas, as well as ERα positive tumors and breast cancer cell lines. LSR levels were significantly reduced in claudin-low breast cancer cell lines and functional studies illustrated that re-introduction of LSR into a claudin-low cell line suppressed the EMT phenotype and reduced individual cell migration. However, our data suggest that LSR may promote collective cell migration. Re-introduction of LSR in claudin-low breast cancer cell lines reestablished tight junction protein expression and correlated with transepithelial electrical resistance, thereby reverting claudin-low lines to other intrinsic molecular subtypes. Moreover, overexpression of LSR altered gene expression of pathways involved in transformation and tumorigenesis as well as enhanced proliferation and survival in anchorage independent conditions, highlighting that reestablishment of LSR signaling promotes aggressive/tumor initiating cell behaviors. Collectively, these data highlight a direct role for LSR in driving aggressive breast cancer behavior.

## Introduction

Breast cancer is a heterogeneous disease that varies in its etiology, pathophysiology and response to therapy. Breast cancer patients with disease of similar stage and grade often respond differently to therapy resulting in disparate clinical outcomes. In attempts to understand the biological and clinical diversity of breast tumors, Perou and colleagues have developed molecular profiles characterizing the various intrinsic breast cancer subtypes, which have been successful at prediction of overall survival, relapse, and response to chemotherapy [1]–[4]. The claudin-low subtype is of particular interest due to its aggressive behavior. Clinically, the majority of these tumors are invasive ductal carcinomas with a triple negative phenotype (lacking the estrogen receptor (ER) and progesterone receptor (PR), and do not overexpress the growth factor receptor Her2). While these tumors initially respond to chemotherapy, there is a high risk of recurrence, disease progression and, consequently, patient survival is poor [5], [6].

The claudin-low subtype is characterized by cancer stem cell-like features and low gene expression of junction and adhesion proteins including claudin 3, 4 and 7 and E-cadherin [3]. Recently, the lipolysis stimulated lipoprotein receptor (LSR) was reported to be highly expressed in cells resistant to chemotherapy *in vitro* and correlated with tumor-initiating capacity *in vivo* using CD44^hi^/24^lo^ epithelioid basal A cells derived from a triple negative cell line [7]. However, the functional role of LSR in breast cancer cell behavior has not been directly investigated.

LSR was originally identified as a hepatocyte receptor and was shown to regulate post-prandial uptake of triacylglyceride-rich lipoproteins [8]. LSR is involved in the dynamics of lipid distribution between the liver and peripheral tissue, is sensitive to high fat diets and is regulated by circulating leptin. Given the emerging role of obesity and altered cellular metabolism in breast cancer [9], and the recent report highlighting the role of LSR in tumor initiating breast cancer cell populations [7], functional studies directly testing the role of LSR in breast cancer cell behavior were conducted.

The levels of LSR were quantified in primary breast tumor biopsies and significant associations were identified when correlated with cancer stage, pathology, and hormone receptor status. LSR levels were significantly associated with specific intrinsic breast cancer molecular subtypes when tested in representative breast cancer cell lines. Furthermore, *in vitro* model systems were used to study the functional role of LSR in breast cancer cell behavior. Our data suggest that expression of LSR may direct collective cell migration and inhibit individual cell migration in breast cancer cells. Overexpression of LSR in claudin-low breast cancer cell lines re-established a family of TJ protein expression, thereby reverting claudin-low lines to other intrinsic breast cancer molecular subtypes. In addition, overexpression of LSR enhanced proliferation and survival in anchorage independent conditions, as well as significantly increased genes reported to be involved in transformation and tumorigenesis. Collectively, these data show a direct role for LSR in promoting aggressive breast cancer behavior.

## Materials and Methods

### Ethics Statement

All patient samples were performed in accordance with the guidelines of the North Carolina Central Review Board, under protocol number 1201027. All samples were analyzed anonymously and were obtained de-identified from the vendor.

### Cell culture

All cell lines were obtained from American Type Culture Collection (ATCC), with the exception of SUM cell lines that were obtained from Asterand. Cells were cultured according to manufactures' recommendations and passaged via trypsinization when near 80% confluence. Primary breast epithelial cells were previously isolated and characterized [10], [11]. Cells were maintained in DMEM/F12 supplemented with 5% horse serum, 10 ug/ml insulin, 500 ng/ml hydrocortisone, 20 ng/ml rhEGF, and 1% antibiotics/antimycotics. 76 N normal breast epithelial cells were a kind gift from Dr. R. Shao (University of Massachusetts Amherst) and maintained as described [12].

### Generation of LSR-overexpressing cell lines

Myc-DDK-tagged ORF clone of *Homo sapiens* LSR, transcript variant 1 was obtained from OriGene Technologies (prod: RC223636). Cells were transfected using TurboFectin 8.0 (prod: R0533; Thermo Scientific) according to manufacturers' instructions. For stable transfection, cells were passaged at 1∶10 dilution into fresh growth medium containing 500–900 ug/ml of G418 (Life Technologies). Control cells were simultaneously transfected with an empty plasmid vector and selected in antibiotics as described above. Clonal cell lines were generated via a single cell plated per well and expanded using the assistance of conditioned media from the parental cell line, in addition to standard culture conditions. Clones were evaluated for LSR expression via western analysis prior to functional assays.

### Immunohistochemistry and Immunocytofluorescence

Immunohistochemistry was performed with appropriate controls as described [13]. Briefly, five μm formalin fixed paraffin embedded tissue arrays (U.S. Biomax Inc.; arrays BR2085a and BR805) were de-paraffinized in xylenes, rehydrated, subjected to antigen retrieval using citrate buffer (DAKO), and staining was performed using the Vectastain Elite ABC System (Vector Laboratories) according to manufacturing instructions. Color was developed with diaminobenzidine peroxidase substrate kit (Vector Laboratories) and cores were counterstained with hematoxylin (Sigma Aldrich). The anti-LSR and anti-ERα antibodies were both used at a 1∶100 dilution (SC-133765; Santa Cruz Biotechnology and NCL-ER-6F11; Lecia). LSR-antibody specificity was confirmed using an additional commercially available antibody validated for immunohistochemistry (*data not shown*, Atlas Antibodies; Stockholm, Sweden, catalogue number HPA007270). Imaging was performed on an Olympus 1×51 microscope and quantified using NIH Image J64 software as previously described [14] (threshold standardized; measurement determined as percent area: red). A total of 143 invasive ductal carcinomas and 105 invasive lobular carcinomas and their associated age/menopause status, presence or absence of ERα, and TNM status were analyzed. ERα expression was determined by both clinical reports with the arrays and via immunohistochemistry. Staining scores were defined as below-detection, weak, and strong staining.

Immunocytofluorescence was performed as we previously described [15]. Briefly, cells were grown on 8-well chamber slides (Research Products International, Mt. Prospect, IL,) and fixed/permeabilized in ice-cold methanol:acetone. Following fixation, cells were blocked in 1% BSA and 5% normal horse serum PBS, stained with the indicated primary antibody (1∶100 dilution) for one hour at 4°C (anti-LSR, SC-133765 and anti-ZO1, SC-8146, Santa Cruz Biotechnology; anti-α-tubulin; T6199, Sigma Aldrich), washed and then incubated for 30 minutes with an anti-rabbit, anti-mouse or anti-goat Alexa Fluor 488 secondary antibody (1∶1000 dilution, Invitrogen). Coverslips were applied with ProLong® Gold Antifade Reagent with DAPI (Life Technologies). Imaging was performed on a Nikon DiaPhot microscope with digital camera and using Spot Advanced software version 4.5 (Sterling Heights, Michigan).

### Western blot analysis

Cells were lysed in RIPA Buffer (50 mM Tris Base, 150 mM NaCl, 1 mM EDTA, 1% NP40, 0.25% sodium deoxycholate) supplemented with protease and phosphatase inhibitors (Halt™ Thermo Scientific). Equal protein concentrations of total cell lysates, as determined by the Coomassie Plus Protein Assay (Thermo Scientific), were separated by SDS-PAGE under reducing conditions. Proteins were transferred to nitrocellulose membranes (BioExpress). Membranes were blocked in 5% non-fat milk in TBST (1.0 M Tris-HCL, 5.0 M NaCl, 0.1% Tween) for 1 hour at room temperature, then incubated with primary antibody (LSR, SC133765; TIAM1 SC827; FLT1 SC316, N-Cadherin SC271386, Santa Cruz Biotechnology; AF6 610732, BD Transduciton Labs; Claudin7 34–1500, Occludin 33–1500, Zymed; PKCζ CST9372, Cell Signaling) overnight at 4°C in TBST containing 5.0% BSA, washed, and incubated with the appropriate secondary antibody conjugated to horseradish peroxidase (GE Healthcare) in TBST with 5% milk for 1 hour at room temperature. Mouse monoclonal α-tubulin antibody was used as a loading control at 1∶5000 dilution (T6199; Sigma Aldrich). Enhanced chemiluminescence detection system (ECL Plus, GE healthcare) was used to detect peroxidase activity. NIH Image J64 was used to quantify western blots.

### Proliferation, Migration, Soft Agar and Tumorsphere assays

For all assays, a minimum of three independent experiments were performed as previously described [11]. Proliferation assays: cells were plated in triplicate for each time point, and at the predetermined concentration for each cell line. Manual cell counts were taken every 24 h for a total of 120 h. Cell cycle analysis: cultured cells in log phase growth were harvested by trypsinization, resuspended in PBS, and fixed with 95% ethanol at 4°C for 24 h. Fixed cells were collected by centrifugation, resuspended in staining solution (PBS, 2 mM MgCl_2_, 10 ug/ml RNase A, 100 ug/ml propidium iodide) and incubated at 37°C for 20 min. Single cell populations (1×10^6^ cells/ml) were analyzed and cell cycle analysis was performed using BD Accuri C6 Flow Cytometer and accompanying software (Becton-Dickinson Bioscience, Franklin Lakes, NJ). Migration assays: Costar transwell permeable support 8.0 um polycarbonate membranes were used according to the manufacturer's protocol. Cells were washed, resuspended in serum-free medium, and plated in the top chamber of transwell inserts (5×10^4^ cells per insert; each line plated in duplicate). The cells were allowed to invade through the membrane for four to 16 h towards 10% FBS-containing medium in the bottom chamber. Following invasion, the cells were wiped from the top surface of the membrane; the remaining cells were fixed in methanol and stained with a 1% toluidine blue solution. Cell numbers were determined from microphotographs taken over four (non-overlapping) areas of the membrane. Migration wound assays: Cells were grown to confluence, a scratch was made with a pipette tip, and the wound was allowed to close for up to 16 h. Phase images were taken every 2–4 h at 10 and 20x. Image J was used to quantify percentage of wound closure by measuring and comparing the area free of cells at 0 hours and at 8 h. Soft agar assays: equal numbers of cells were plated, in triplicate, on an 0.66% agarose base in a 0.33% top soft agar layer in 35 mm cell culture dishes. Cells were incubated for 7 days and then stained overnight with nitrobluetetrazolium. The total number of colonies in each well was counted. Tumorsphere assays: cells were cultured as published [16]. Briefly, cells were seeded into 24-well ultralow attachment plates (Corning Life Sciences) containing 500 uL of DMEM/F12 (Invitrogen) supplemented with 40 ng/mL human epidermal growth factor (PeproTech), 20 ng/mL basic fibroblast growth factor (PeproTech), and 2.0% B27 (Invitrogen). Cells were sorted in the following dilutions: 50, 100, 250, 500, and 1000 cells per well. Cells were cultured at 37°C for 10 days. Tumorsphere growth was quantified microscopically under 20x magnification.

### Transepithelial electrical resistance (TER)

Cells were plated on Costar® 0.4 μm Polycarbonate membrane Transwell® 6-well plates and grown to confluence. Two days post a high-density monolayer had formed the TER was measured directly in culture medium using an epithelial volt-ohm meter (EVOM2, World Precision Instruments). A minimum of three independent experiments, each measured in triplicate. Results are presented as Ω ⋅ cm^2^. Immediately following final TER reading, cells were directly lysed on the transwell insert in lysis buffer and subjected to western analysis as described above.

### Quantitative Real Time PCR and PCR Arrays

Total RNA was isolated from proliferating cells using the RNeasy kit (Qiagen) according to the manufacturer's instructions. RNA was reverse transcribed using MMLV reverse transcriptase (Invitrogen) and primed with oligo-dT and random hexamers (Invitrogen). CDbGeo and pTD cDNA was amplified using gene-specific primers for murine LSR (5′-AGGCTAACCAGCAAGATGACTCCA-3′, 3′-AGGTTACTTCACTCATGGCCCGTT-5′) GAPDH [10], SLUG and SNAIL [17]. For PCR arrays, RNA was reverse transcribed using the RT^2^ Frist Strand Kit and RT^2 Profiler^ PCR arrays (Tight Junction; PAHS-143Z, Breast Cancer PAHS-131Z, Qiagen) according to the manufacturer's instructions. Data were analyzed via the comparative CT (^ΔΔ^CT) method and using the online analysis tools provided (Qiagen).

### Statistical analysis

Tumor pathology, size, presence of metastasis to primary lymph node, and metastatic staging compared to intensity of LSR staining was evaluated for the significance via one-way analysis of variance (ANOVA) with the Bonferroni multiple comparisons or Mann-Whitney *post hoc* analysis, or *t* tests using GraphPad Prism 6.0 (GraphPad Software). Age and Menopausal status compared to intensity of LSR staining was also carried out via *t* tests or ANOVA. ERα status in relation to LSR staining was visualized by NIH Image J64 and measured by GraphPad Prism 6.0. Data was considered significant at *P*<0.05. All histograms were generated in GraphPad Prism as mean ± S.E.M. Graphs were plotted in Microsoft Excel as mean ± S.D or SEM as indicated.

## Results

### LSR protein expression in breast tissue, breast tumor biopsies and correlation with clinical variables

**Figure 1:**
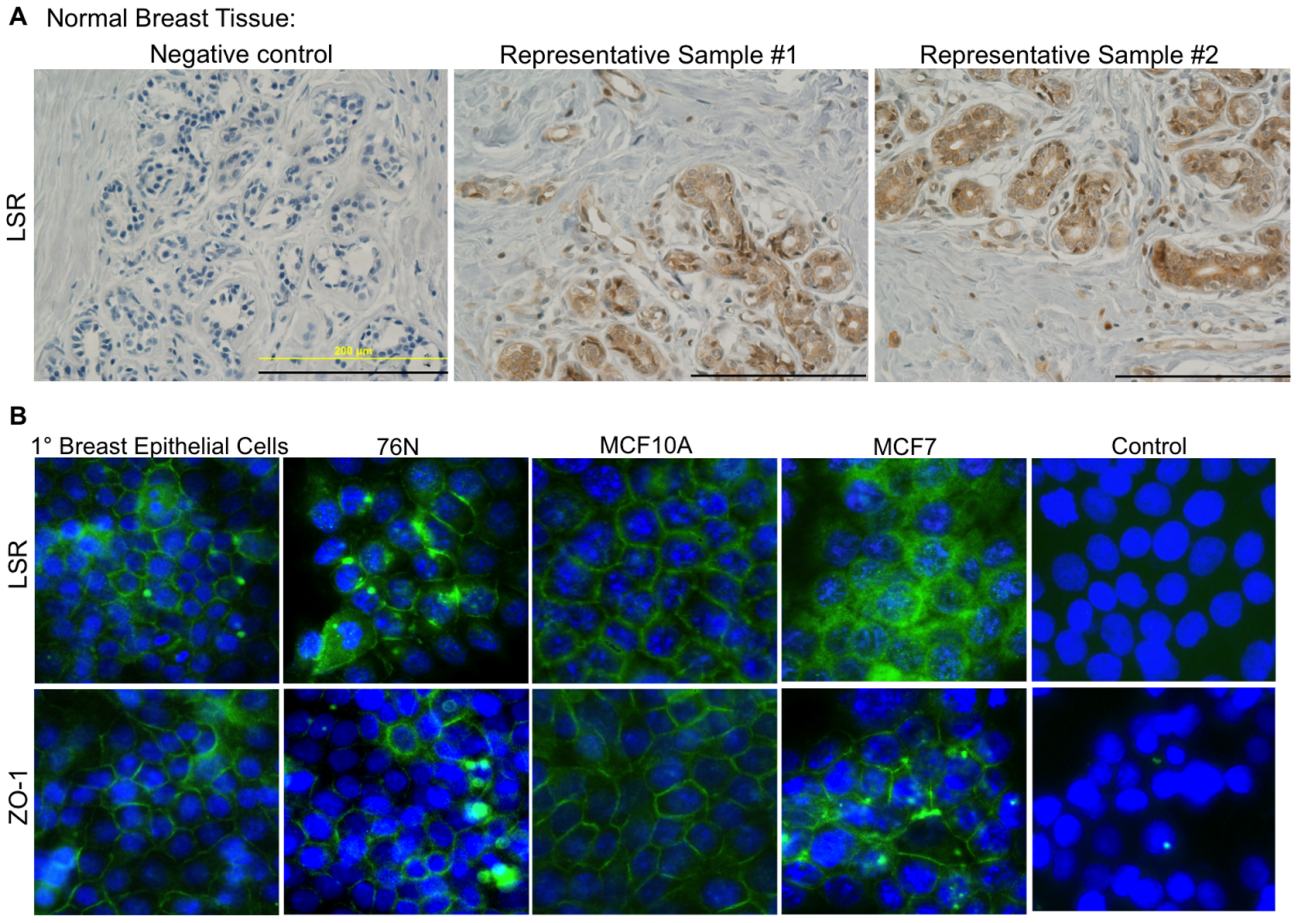
LSR protein expression in breast tissue and epithelial cells. (A) Representative images of normal breast tissue arrays subjected to immunohistochemical analysis using a LSR specific antibody or corresponding negative control. Scale bar = 200 uM. (B) Representative images of breast epithelial cells subjected to immunocytofluorescence using LSR and ZO-1 specific antibodies (DNA stained with DAPI). Control images are primary breast epithelial cells simultaneously subjected to all steps with the exception of the addition of the primary antibody. Images were obtained at 20X.

We began our investigation by evaluating LSR protein expression levels in breast epithelial cells, breast tissue, and breast cancer biopsies. LSR was readily detectable in the normal breast epithelium and endotheilum, as well as evident in immune cells and stromal tissue via immunohistochemistry (Fig. 1A). To further detail the localization of LSR in the breast epithelium, immunocytofluorescence was performed on primary breast epithelial cells and normal breast epithelial cell lines (Fig. 1B). MCF7 cells were used as a comparison of LSR localization in cancer cells to normal breast epithelia cells and ZO-1 was used as a control to illustrate membrane localization. Similar to results in breast tissue histosections, LSR was primarily localized to the cell membrane in all cells evaluated. It is of note that MCF7 cells appeared to have a more diffused localization and higher abundance of cytoplasmic LSR compared to normal breast epithelium.

**Figure 2:**
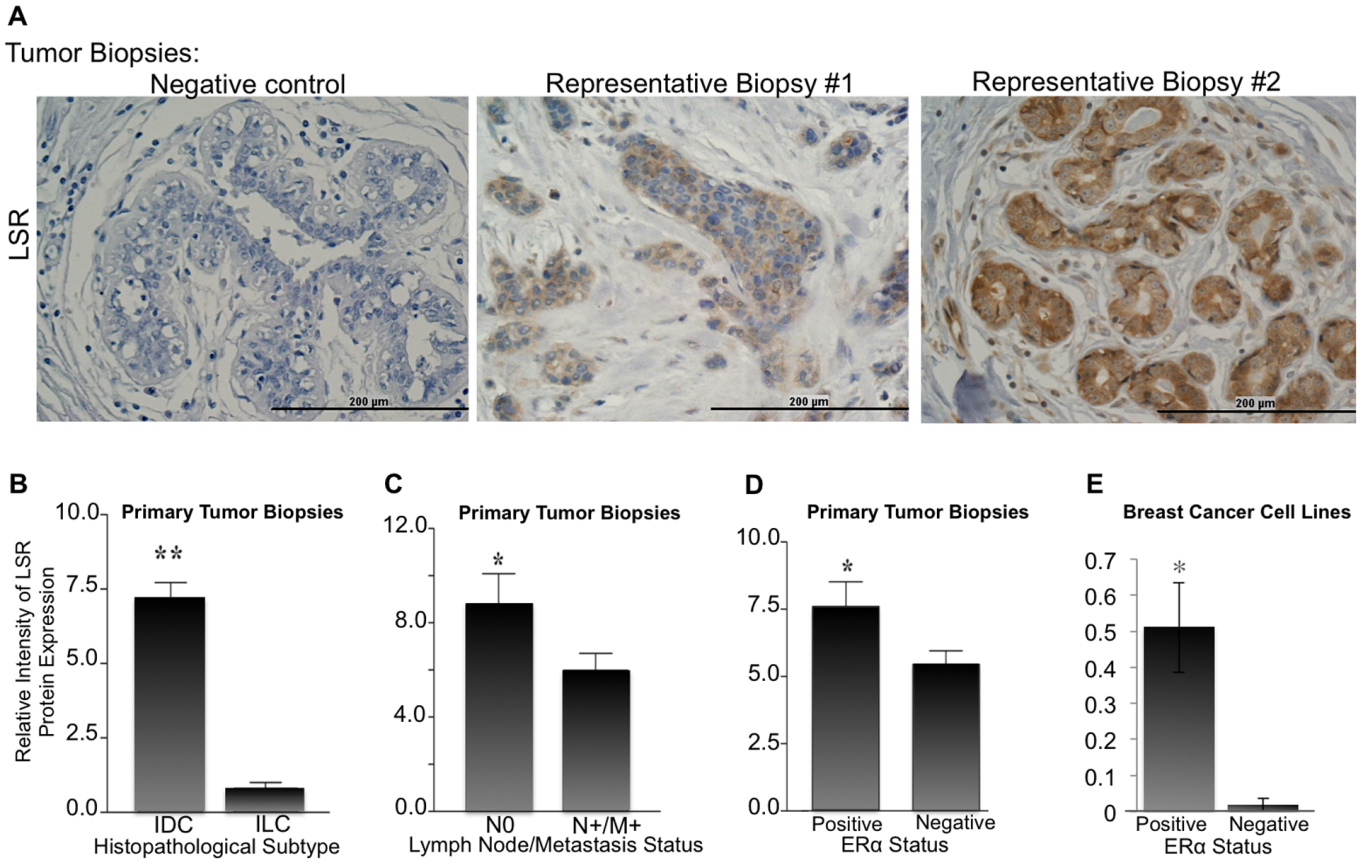
LSR protein expression in breast biopsies and correlation with clinical variables. (A) Representative images of breast cancer biopsy tissue arrays subjected to immunohistochemical analysis using a LSR specific antibody or corresponding negative control. Scale bar = 200 uM. Intensity of LSR expression in correlation with (B) breast cancer subtype IDC  =  invasive ductal carcinoma, ILC  =  invasive lobular carcinoma, (C) tumor invasion into sentinel lymph node and/or distant metastasis, (C) ERα status in tumor biopsy, and (D) ERα status in ERα positive breast cancer cell lines (MCF7, ZR75-1, and T47D) and ERα negative breast cancer cell lines (MDA-MD-231, SUM159, Hs578t). Data represent mean relative intensity +/− SE. *P<0.05, **P<0.01. A total of 248 patient samples were analyzed.

The relative intensity of LSR expression in the tumor epithelium distinctly varied among patient biopsies (Fig. 2A). To evaluate if the changes in LSR protein levels correlated with clinical variables, the pateint samples were classifed into three different populations based on the relative intensity of LSR expression (below-detectable, weak, or strong) levels based on threshold values; Fig. 2B biopsy #1 and #2, respectively for weak and strong staining. Quantification was determined using NIH Image J64 software (threshold standardized; measurement determined as percent area: red). Analysis of 248 breast biopies showed that high expression levels of LSR were significantly correlated with invasive ductal carcinomas (IDC) compared to invasive lobular carcinomas (ILC, Fig. 2B, *P*<0.01). No correlation was found between LSR expression and tumor size, age, or menopausal status of the patients (Fig. S1). However, LSR expression was found to be significantly reduced in patient samples with lymph node invasion and distant metastasis (Fig. 2C, *P*<0.05).

ERα expression is known to be present in both IDC and ILC, and the presence or absence of ERα often dictates treatment regimen [18], [19]. We next sought to correlate ERα expression with LSR in tumor biopsies. ERα was determined via the provided pathological data from each patient sample and verified via immunohistochemistry (Fig. S2). A significant correlation was found between tissues expressing both high levels of ERα and LSR (Fig. 1D, *P*<0.005). In addition, we used representative ERα positive and negative breast cancer cell lines to confirm our results observed with the tumor biopsies. LSR levels were analyzed via western analysis, and similar to tumor biopsies, a significant correlation was observed between ERα positive cell lines and high LSR expression (Fig. 2E *P*<0.001).

### LSR expression in breast cancer molecular subtypes

**Figure 3:**
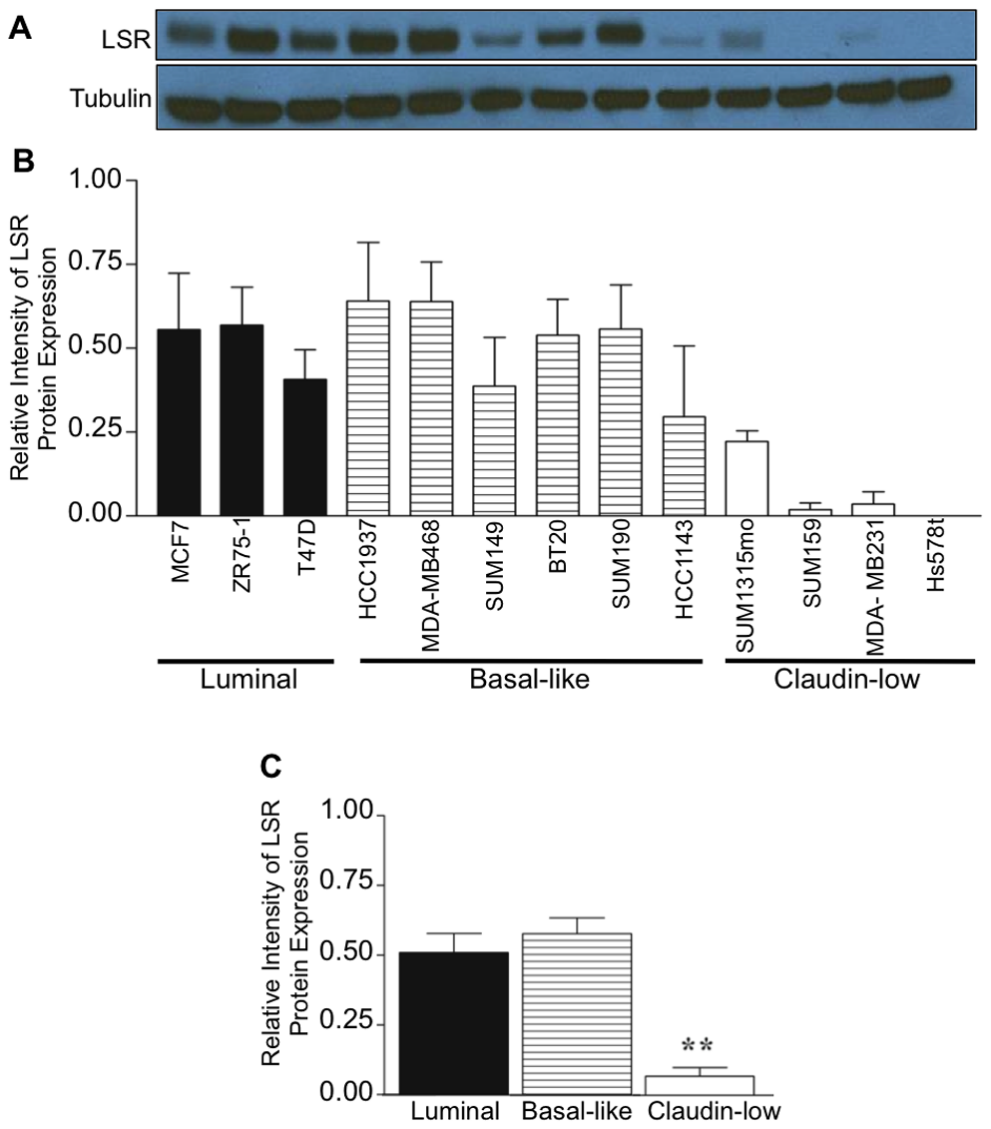
LSR expression in breast cancer molecular subtypes. Representative breast cancer cell lines were grown to 80% confluence, lysates were isolated and analyzed via western analysis using a LSR specific antibody; α-tubulin was used as a loading control. (A, B) Representative western blot and corresponding intensity measured via ImageJ. (C) Analysis based on molecular subtype of cell lines. Data represent mean relative intensity +/− SE. **P<0.01.

One of the hallmarks of claudin-low molecular subtypes of breast cancer is the absence of ERα, as well as other luminal differentiation markers, and our data suggests that LSR is positively correlated with ERα expression. Thus, we hypothesized that LSR would be lost in claudin-low molecular subtypes. To test this hypothesis, a comprehensive panel of well-characterized cell lines from the representative molecular subtypes was analyzed for LSR expression via western analysis (Fig. 3). LSR was readily detectable in the majority of luminal and basal-like cell lines; however, the claudin-low lines evaluated had significantly lower LSR expression compared to luminal and basal-like classified cell lines (*P<0.01). Hs578t, a highly aggressive and metastatic cell line [20], had no detectable levels via western analysis. Collectively, these data support our tumor biopsy data in that LSR expression varies by subtype, with decreased levels in subtypes associated with poor prognosis.

### Reintroduction of LSR into a claudin-low breast cancer cell line and the resultant effect on cell behavior

**Figure 4:**
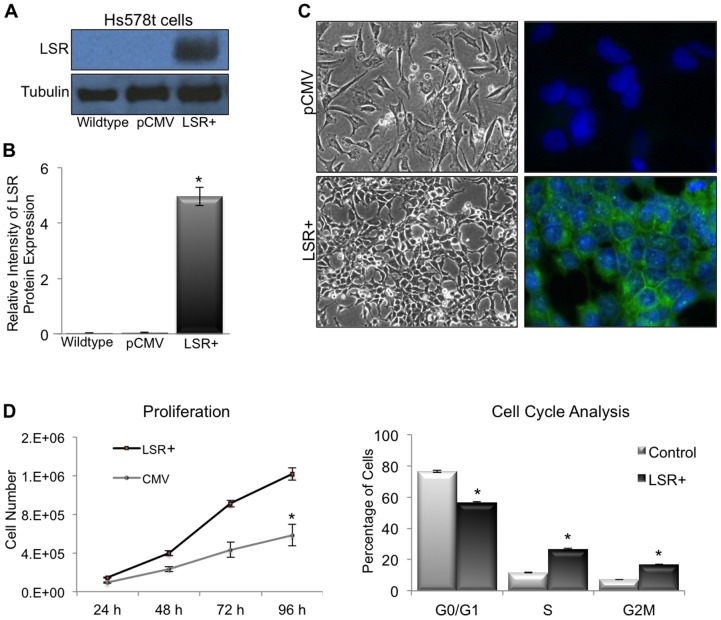
LSR expression in a claudin-low breast cancer cell line. Hs578t cells were stably transfected with either a control plasmid (pCMV), or a plasmid containing the full-length gene for LSR variant 1 (LSR+). Cell lines were grown to 80% confluence; lysates were isolated and analyzed via western analysis using and LSR specific antibody and α-tubulin for loading control. Intensity measured via ImageJ. (A) Representative western blots. (B) Data represent mean relative intensity +/− SE. *P<0.001. (C) Representative images of cells growing on tissue culture treated dishes (left panels; 20× magnification) and cells subjected to immunocytofluorescence using a LSR specific antibody (right panels; DNA stained with DAPI, 40× magnification). (D) Proliferation assays. Cells were plated at 50,000 cells per well in triplicate and counted every 24 h for 96 h. Data represent mean +/− SE. *P<0.001. (E) Cell cycle analysis: cells growing in log-phase were fixed and stained with propidium iodine and analyzed via flow cytometry. Data represent mean +/− SE. *P<0.03.

To begin to understand the role of LSR in directing cell behavior, we reintroduced LSR into the Hs578t claudin-low breast cell line (LSR+) that had undetectable levels of LSR via western blot analysis (Fig. 4A, B). Cell morphology was noticably changed upon confirmed expression of LSR; the overall cell size of LSR+ cells appeared reduced, and LSR+ cells grew in distinct clusters compared to pCMV control cells (Fig. 4C) when plated at similar densities/equivalent cell numbers.

The localization of LSR expression was predominatly on the cell membrane, however, similar to the pattern in the MCF7 cancer cell line (Fig. 1B) LSR was also prominent in the cytoplasm. Interestingly, reintroduction of LSR resulted in a signifincat increase in proliferation rate compared to control cells (Fig. 4D, left; *P<0.001). Cell cycle anlaysis revealed that LSR+ cells had a shift in cell cycle towards S and G_2_/M phase compared to control cells (Fig. 4D, right; *P*<0.02). Correspondingly, LSR+ cells had a significant decrease in the percentage of cells in the G_0_/G_1_ phase (*P*<0.03). Together these data suggest reintroduction of LSR expression in Hs578t claudin-low cells alters cell behavior, including cell morphology and proliferation.

**Figure 5:**
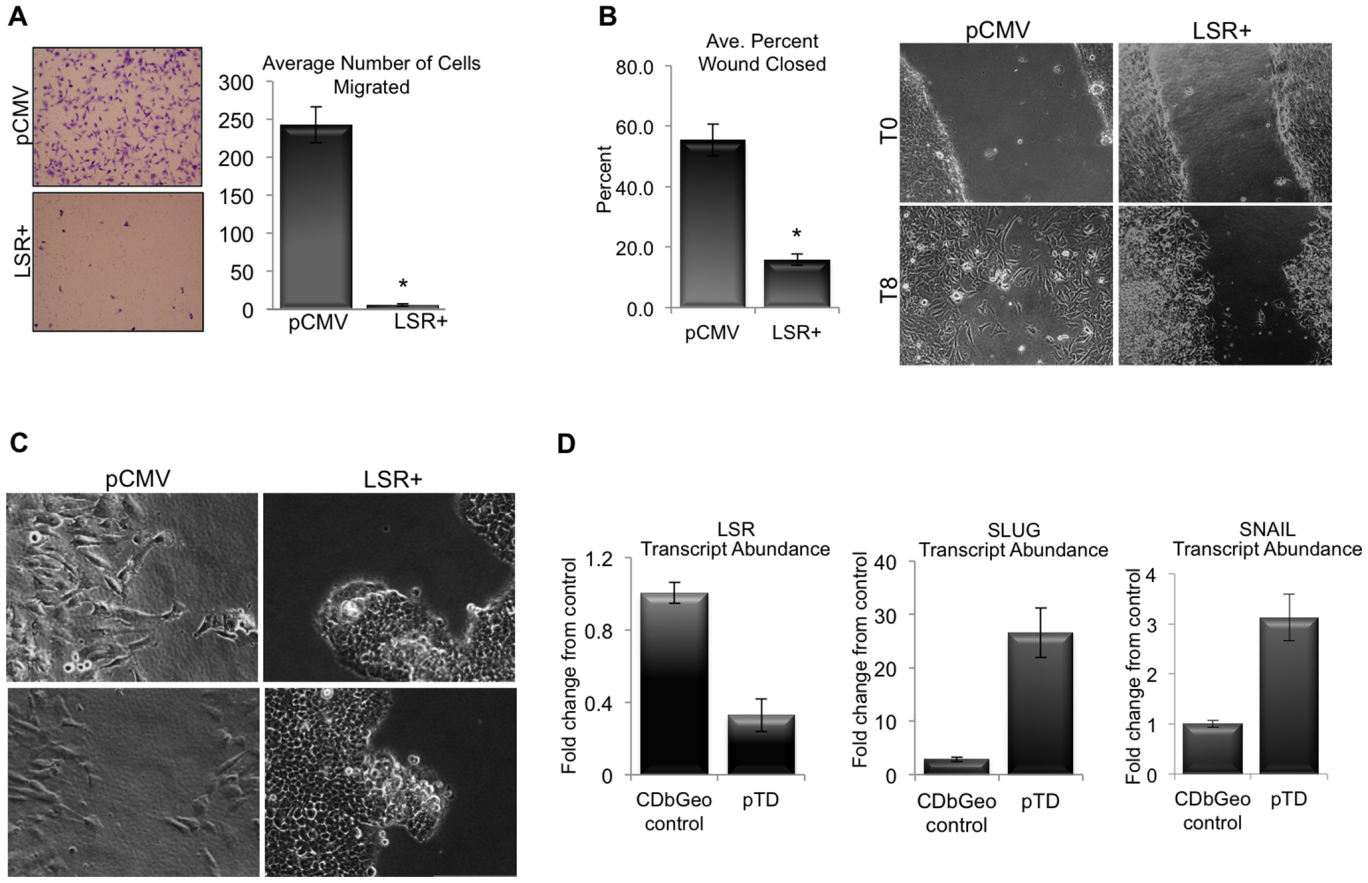
Effect of LSR expression on breast cancer migratory cell behavior. Hs578t cells were stably transfected with either a control plasmid (pCMV), or a plasmid containing the full-length gene for LSR variant 1 (LSR+). (A) Cell migration: Cell were allowed to migrate through transwell inserts towards media containing 10% serum for up to 16 h then fixed, stained and counted (left; representative image of migrated cells, right; quantitation). (B) Wound assay: Cells were grown to confluence, and then a scratch was induced down the middle of the monolayer. Cells were imaged at every four h for 16 h. Data represent mean +/− SE of percent wound closure at eight h (10X). (C) Representative images of cell behavior during wound closure at 20X. (D) Quantitative RT-PCR analysis of validated cell line model system that comparing control (CDbGeo) and the terminally differentiated EMT phenotype (pTD) for LSR expression. SNAIL and SLUG were evaluated to confirm EMT.

We next performed functional assays to direclty test the effect of LSR on breast cancer cell behavior. Although increased proliferation suggested a more aggressive cell behavior, LSR expression caused a signficant decrease in the ability of individual cells to migrate through a porous membrane towards a chemoattractant (Fig. 5A; *P<0.001). In addition, wound healing scratch assays revealed an altered capacity for cells to migrate and close a wound depending on the expression of LSR, as LSR+ cells closed the induced scratch at a slower rate compared to control cells (Fig. 5B; *P<0.01). Noteably, the phenotype and migratory behavior of the LSR+ cells during wound healing was indicative of collective cell migration. The control cells closed the scratch via individual cells migrating into the wound, while the LSR+ cells moved as a collective whole, budding into the wound (Fig 5C). The phenotype is similar to the morpholgy observed during the migration of the terminal ductal lobular structures into the adipose tissue during pubertal development [21].

Thus, to further investigate the role of LSR in migratory behavior we utilized a validated epithelial-to-mesenchymal (EMT) mammary cell line system to observe the relative expression of LSR in both the epithelial and mesenchymal phenotypes of the same parental cell line (CDbGeo and pTD for “persistently trans-differentiated cells”, respectively) [17]. As shown in Fig. 5D, the pTD that have been transformed by TGFβ, and exhibit a mesenchymal phenotype, express significantly lower LSR levels compared to control cells. EMT was confirmed by increases in SLUG and SNAIL in the TGFβ stimulated cells. Collectively these data suggest that the expression of LSR reduces individual migratory cell behavior. However, the enhanced proliferative capacity and collective migration of the cells warranted further analyses.

**Figure 6:**
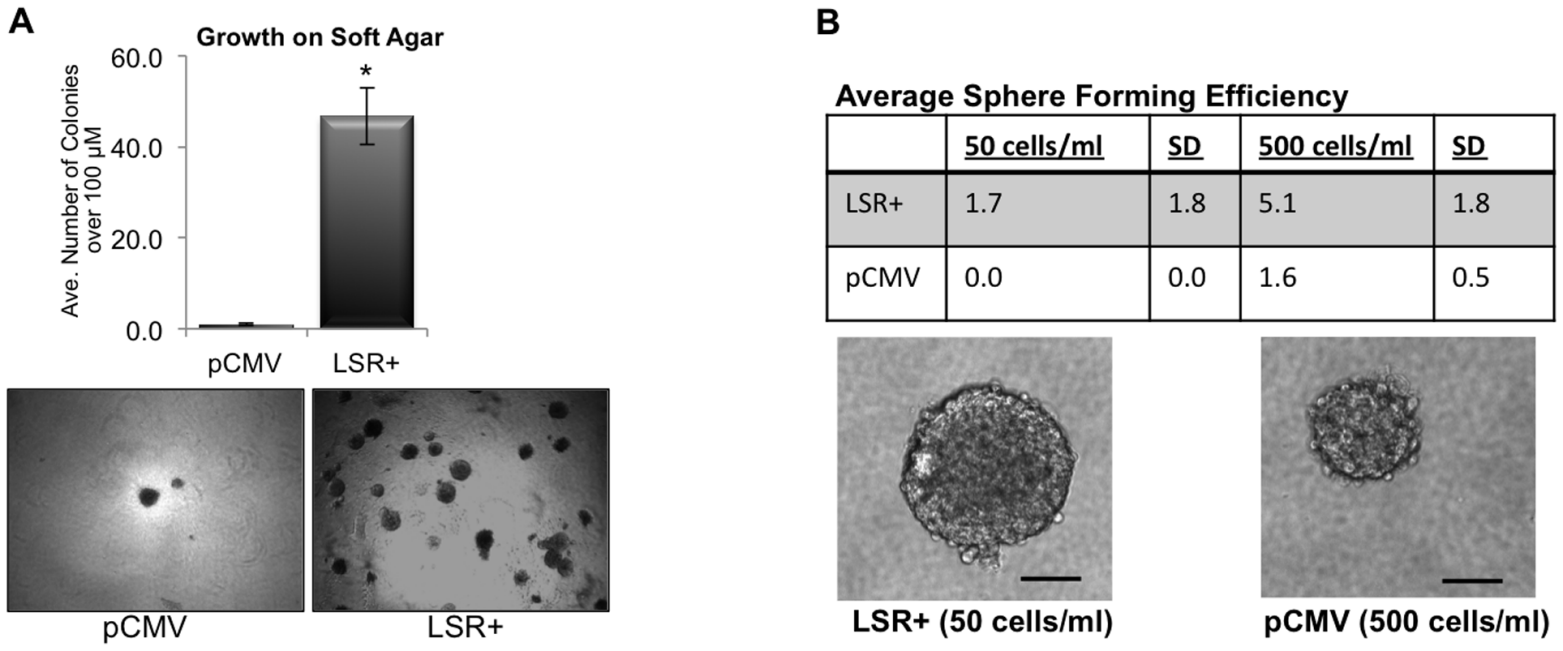
LSR enhances cell survival in non-adherent culture conditions. Hs578t cells were stably transfected with either a control plasmid (pCMV), or a plasmid containing the full-length gene for LSR variant 1 (LSR+). (A) Soft agar assays. Cells were plated on soft agar coated wells, grown for seven days, then stained with nitrobluetetrazolium before counting. The entire dish was analyzed and colonies larger than 50 um in diameter were counted. Data represent mean colonies counted per well ± SE of three separate experiments; *P<0.001. Bottom panels are representative images at 10X. (B) Sphere forming efficiency. Cells were plated in DMEM +10 ng/ml EGF +20 ng/ml FGF +2% B27 in ultralow attachment dishes for seven days then spheres counted and imaged. Data represent mean +/− SD of three independent experiments at the indicated cell plating densities. Bottom panels are representative phase images of anchorage-independent, single-cell derived spheres from LSR+ and control pCMV cells after seven days of growth. Scale bar, 50 *u*m.

We challenged the pCMV and LSR+ cells to grow in anchorage-independent conditions, thereby mimicking changes that occur during tumorigenesis. Soft agar transformation assays revealed that expression of LSR significantly enhanced the ability of individual cells to survive and form colonies on soft agar (Fig. 6A, *P<0.001). Next, sphere formation assays were performed to determine if the expression of LSR enhanced survival, self-renewal, and growth in suspension culture. Results show that individual LSR+ cells were able to form tumorspheres at densities as low as 50 cells/ml compared to control cells which only formed sphere starting at 500 cells/ml (Fig. 6B). Corresponding with our results of enhanced proliferation of LSR+ cells on tissue culture dishes, we observed that the spheres formed from the LSR+ cells were larger in diameter compared to spheres formed from pCMV cells after seven days of growth.

To rule out clonal variabilty, three clonal cell lines with varying levels of LSR expression were established and tested (Fig. S3). Similar to results with polyclonal colonies, LSR+ clones had reduced cell size and altered morphology, enhanced proliferation, reduced individual cell migration, and enahnced survival and self-renewal/growth in non-adherent condtions compared to controls. The intensity of the observed cell behaviors corrrelated with LSR expression levels in a dose dependent manner; the higher the expression of LSR the more robust the cellular response.

**Figure 7:**
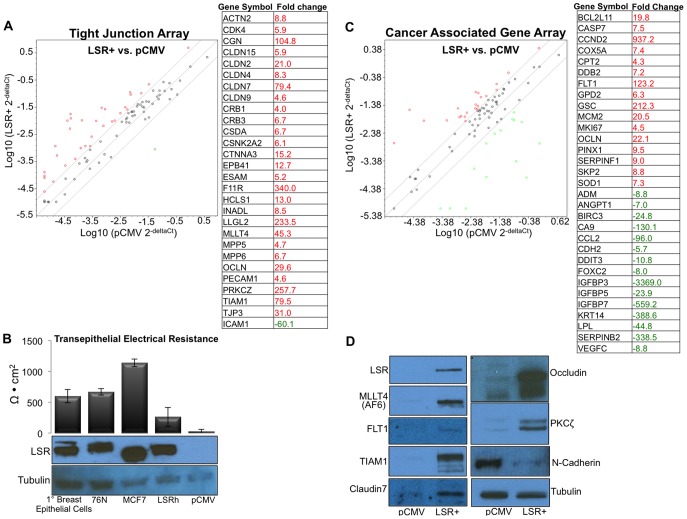
Re-introduction of LSR alters tight junction and breast cancer related gene expression. Hs578t cells were stably transfected with either a control plasmid (pCMV), or a plasmid containing the full-length gene for LSR variant 1 (LSR+). Quantitative real-time PCR analysis was performed for genes associated and with tight junctions and adhesion proteins (A). Data show representative scatterplots and corresponding list of genes significantly altered by expression of LSR. (B) Transepithelial Electrical Resistance in breast epithelial cells and breast cancer cells +/− LSR expression. Cells were plated on transwell inserts and grown to confluence. Two days post formation of a high-density monolayer TER was measured directly in culture medium. Cell monolayers were directly lysed on the transwell insert following final TER measurement and subjected to western analysis using an LSR specific antibody. Tubulin was used as a loading control. Date represent a minimum of three independent experiments, each measured in triplicate. (C) Quantitative real-time PCR analysis was performed for genes associated and with deregulation in cancer. Data show representative scatterplots and corresponding list of genes significantly altered by expression of LSR. (D) Representative western blots highlighting protein level changes of a panel of genes found to be differentially expressed in the arrays.

Given the dramatic phenotype and behavioral changes observed with overexpression of LSR in a claudin-low cell line, we performed a pathway targeted PCR array analyses to gain further insight into the global gene expression changes upon reintroduction of LSR. Results show that reintroduction of LSR was able to collectivley reestablish the expression of a significant number of tight junction, cell adhesion, and cytoskeletal-activity related genes (Fig. 7A). Thus, we investigated whether LSR expression correlated with barrier function in the LSR+ cell lines and normal breast epithelal cells. Monolayers of cells plated on transwell inserts were tested two days post reaching high-density confluence. MCF7 cells served as a positive control as they are known to exhibit high levels of transepithelial electrical resistance (TER) [22]–[24]. Both primary breast epithelial cells and normal breast cancer cell lines demonstrated TER, though the levels were reduced compared to the MCF7 cells (Fig 7B). LSR+ cells demonstrated lower, but detectable level of TER while pCMV control cells had no detectable levels of TER. The level of LSR was comparable between MCF7 and normal breast epithelial cells; however, the shift in molecular weight may suggest different variant or post-translational modifications of LSR. Wildtype Hs57st and SUM159 cells also had no detectable levels of TER (*data not shown*), consistent with their claudin-low subtype and lack of LSR expression (Fig. 3A). These data suggest that re-introduction of LSR may promote an overall differential activation of cytoskeletal/membrane protein transcriptome.

Analysis of the cancer-targeted PCR array similarly show an increase in cytoskeletal and adhesion genes (Fig. 7C). In addition, we observed an increase in several cell cycle genes (Cyclin D2, PIN2, MCM2, CDK4) confirming our observation that LSR+ cells had increased cell prolieration (Fig. 4D). It is of note that the expression levels of several cellular metabolism genes were significanly altered including LPL and CPT1, a protein that functions in mitochondria oxidation of long chain fatty acids. Moreover, in accord with our observations on suppression of EMT in LSR+ cells, a decrease in N-cadherin, angiopoietin-2, IGFBPs, CCL2, FOXC2, VEGFC, and keratin 14 transcript abundance was observed in LSR+ cells.

To confirm results from the arrays, a selection of differentially regulated genes were analyzed via western blot analysis to observed changes in protein expression between the two cell lines. As show in Fig. 7D, the pattern of protein regulation in the LSR+ cells compared to controls followed the results in the array with all proteins tested. Collectivley, these data highlight a collection of pathways for future analysis of LSR function in breast cancer.

## Discussion

In the current study, we show a funtional role for LSR in directing breast cancer cell behavior. Collectively, our breast cancer biopsy analyses suggest that LSR expression is correlated with a less aggressive tumor phenotype. Indeed, our functional studies illustrated that that reintroduction of LSR into a highly invasive cell line suppressed the EMT phenotype, as well as in an independent mammary cell culture model of EMT, and that LSR expression reduced cell migration *in vitro*. However, while expression of LSR reduced the EMT phenotype in breast cancer cells, the high rate of proliferation, escape from anoikis, and the observed collective cell migration behaviors of LSR-containing cells suggests that tumors containing LSR may indeed display an aggressive cancer phenotype. The requisite i*n vivo* studies are currently being performed in our laboratory, however, a recent study evaluating the functional hetergeneity of breast cancer stem-like cells correlated high levels of LSR in breast cancer cells with tumor initiating properties [7]. The authors used a triple-negative breast cancer cell line with a known bi-lineage phenotype to isolate single cells containing high levels of CD44 that exhibited mesenchymal/basal B and luminal/basal A features, respectively. They show that rather than the CD44^hi^/CD24^−^ mesenchymal-like basal B cells, the CD44^hi^/CD24^lo^ epithelioid basal A cells retained classic cancer stem cell features such as tumor-initiating capacity *in vivo*, mammosphere formation and resistance to standard chemotherapy. These tumor-initiating cells also correlated with higher expression levels of LSR compared to mesenchymal/basal B cells.

Our present study complements these findings and extends the research to show direct functional mechanisms of LSR on cell behavior. We similarly observed that LSR containing cells are more epithelioid and have an increased ability to self-renew, survive and grow in anchorage independent conditions. We also directly show the alterations in cell behavior are recapitulated when LSR is reintroduced in a triple-negative cell line. Interestingly, we found that LSR expression significantly correlated with ERα expression in breast cancer biopsies as well as decreased metastasis, while the CD44^hi^/CD24^lo^ study used LSR in their 31 prognostic gene signature to predict distant metastasis [7]. In the present study, we did not find any correlation between LSR expression and distant metastasis in ERα negative tumor biopsies (*data not shown*), suggesting that a multiple gene signature is a more faithful predictor for metastasis.

Reports by Furuse and colleagues have recently shown a distinct, tricellular localization of LSR in the EpH4 murine mammary clonal cell line and other non-mammary epithelial tissues [25]–[28]. Our data show in the normal breast epithelial cells, non-transformed breast epithelial cell lines, as well as the polyclonal and clonal LSR+ cells exhibited a less defined pattern, with LSR localization to the membrane and even found in the cytoplasm, as opposed to only distinct tricellular tight junctions. The method of visualization or selection of the clonal cell lines/generation of knockdown and rescue EpH4 cells may reflect one possibility of these differential observations. However, two recent reports support our data showing that LSR localizes to regions other than tricellular tight junctions in the endothelium of various tissues [28], [29]. Our array data also show a set of signaling proteins reestablished upon LSR overexpression, suggesting the potential of additional cellular functions directed by activation of LSR.

Consistent with other reports, our data show the expression of LSR was correlated with increased barrier function. However, our data suggest this correlation may be due to the role of LSR in re-establishing the expression of tight junction and adhesion proteins, as we do not directly show LSR modulating barrier function or specific tight junction localization. While additional studies are warranted, the possibility exists that tissue specific factors may promote a more generalized membrane-signaling role for LSR in breast tissue, and that activation of LSR signaling drives a re-programming of the transcriptome potentially through the downstream activation of transcription factors. Indeed LSR has been shown to bind to 14-3-3s in HEK293 cells using affinity capture and proteomic analysis [30], and was shown to directly bind lactoferrin in ligand blot assays [31] highlighting a multitude of signaling possibilities for LSR in a tissue specific context.

LSR was originally identified as a hepatic receptor involved in the regulation of postprandial lipemia [32]. In hepatic tissue, LSR undergoes conformational changes upon activation by free fatty acids, thereby revealing binding sites for apoB and apoE proteins and promoting endocytosis [8], [32]–[34]. We observed changes in several metabolism-related genes upon reintroduction of LSR including glucose-6-phosphate dehydrogenase, carnitine palmitoyltransferase 2, and LPL (Fig. 6). Given the hepatic role in of LSR in lipid endocytosis, it is possible that LSR expression altered the cellular metabolism, allowing for the observed enhanced proliferation. It is well known that highly proliferative cancer cells undergo fundamental changes in metabolism, with increased glucose update and glycolysis [35]–[38]. Moreover, changes in the lipid profile of a cell drastically affect cellular metabolism and signal transduction. In relation to cancer, upregulation of lipid metabolism is often observed during the early stages of neoplasia and is a recognized hallmark of many types of cancer [39]. A future goal of our research is to delineate the alterations in breast cancer cellular metabolism with varying levels of LSR expression. Lastly, elevated postprandial lipemia is often associated with obesity, a confounding factor in the development and progression of breast cancer. The obesity-linked adipokine, leptin, is a well-known mitogen/survival factor in breast cancer cells [40], and has been shown to upregulate hepatic LSR levels and ultimately control of hepatic uptake of lipids *in vivo* [34]. It would be of interest to explore whether metabolic status affects levels of LSR in extra-hepatic tissues, including the breast as well as the intracellular changes that occur upon LSR expression.

In conclusion, our data illustrate novel insight into the multifaceted role of LSR in directing breast cancer cell behavior. We show that reintroduction of LSR into breast cancer cells is able to stimulate the expression of genes involved in transformation and tumorigenesis, a family of tight junction/cell adhesion proteins, as well as enhance cellular proliferation and survival in anchorage independent conditions. The reintroduction of LSR into claudin-low breast cancer cells presents a unique model system to study cancer-stem cell characteristics, metabolic driven changes in breast cancer behavior, as well as the intercellular mechanisms that drive the transition between breast cancer molecular subtypes.

## Supporting Information

Figure S1 **LSR protein expression in breast biopsies and correlation with clinical variables.** Breast cancer biopsy tissue arrays were subjected to immunohistochemical analysis using a LSR specific antibody or corresponding negative control. Intensity of LSR expression in correlation with (A) tumor grade, (B) patient menopausal status (premenopausal age 18 to 44 peri-menopausal range 45–53; [41]), and (C) age. Data represent mean relative intensity +/− SE. *P<0.05, **P<0.01. A total of 248 patient samples were analyzed.
(TIF)

Figure S2 **ERα protein expression in breast biopsies.** Breast cancer biopsy tissue arrays were subjected to immunohistochemical analysis using an ERα specific antibody or corresponding negative control. Scale bar = 200 uM. A total of 248 patient samples were analyzed.
(TIF)

Figure S3 **LSR+ Clonal Cell Lines and Functional Assays.** Hs578t cells were stably transfected with either a control plasmid (pCMV), or a plasmid containing the full-length gene for LSR variant 1 (LSR+). Clonal cell lines were generated via a single cell plated per well and expanded using the assistance of conditioned media from the parental cell line, in addition to standard culture conditions. (A) Western analysis confirmation of LSR expression. (B) Representative images of immunocytofluorescence using a LSR specific antibody (DNA stained with DAPI). (C) Proliferation assays: cells were plated at 50,000 cells per well in triplicate and counted every 24 h for 96 h. Data represent mean +/− SD. *P<0.01. (D) Sphere forming efficiency: cells were plated in DMEM +10 ng/ml EGF +20 ng/ml FGF +2% B27 in ultralow attachment dishes for seven days then spheres counted and imaged (scale bar, 50 *u*m). (E) Soft agar assays: cells were plated on soft agar coated wells, grown for seven days, and then stained with nitrobluetetrazolium before counting. The entire dish was analyzed and colonies larger than 50 um in diameter were counted. Data represent mean colonies counted per well ± SD; *P<0.001. Top panels are representative images at 20X. (F) Cell migration: Cell were allowed to migrate through transwell inserts towards media containing 10% serum for up to 16 h then fixed, stained and counted (top; representative image of migrated cells, bottom; quantitation). Data represent mean number of cells counted per field ± SD; *P<0.001.
(PDF)
