# Supplementary figures and images for: The Role of Lipolysis Stimulated Lipoprotein Receptor in Breast Cancer and Directing Breast Cancer Cell Behavior
Source: PLoS One. 2014 Mar 17;9(3):e91747. doi: 10.1371/journal.pone.0091747 (PMC3956714; doi:10.1371/journal.pone.0091747)

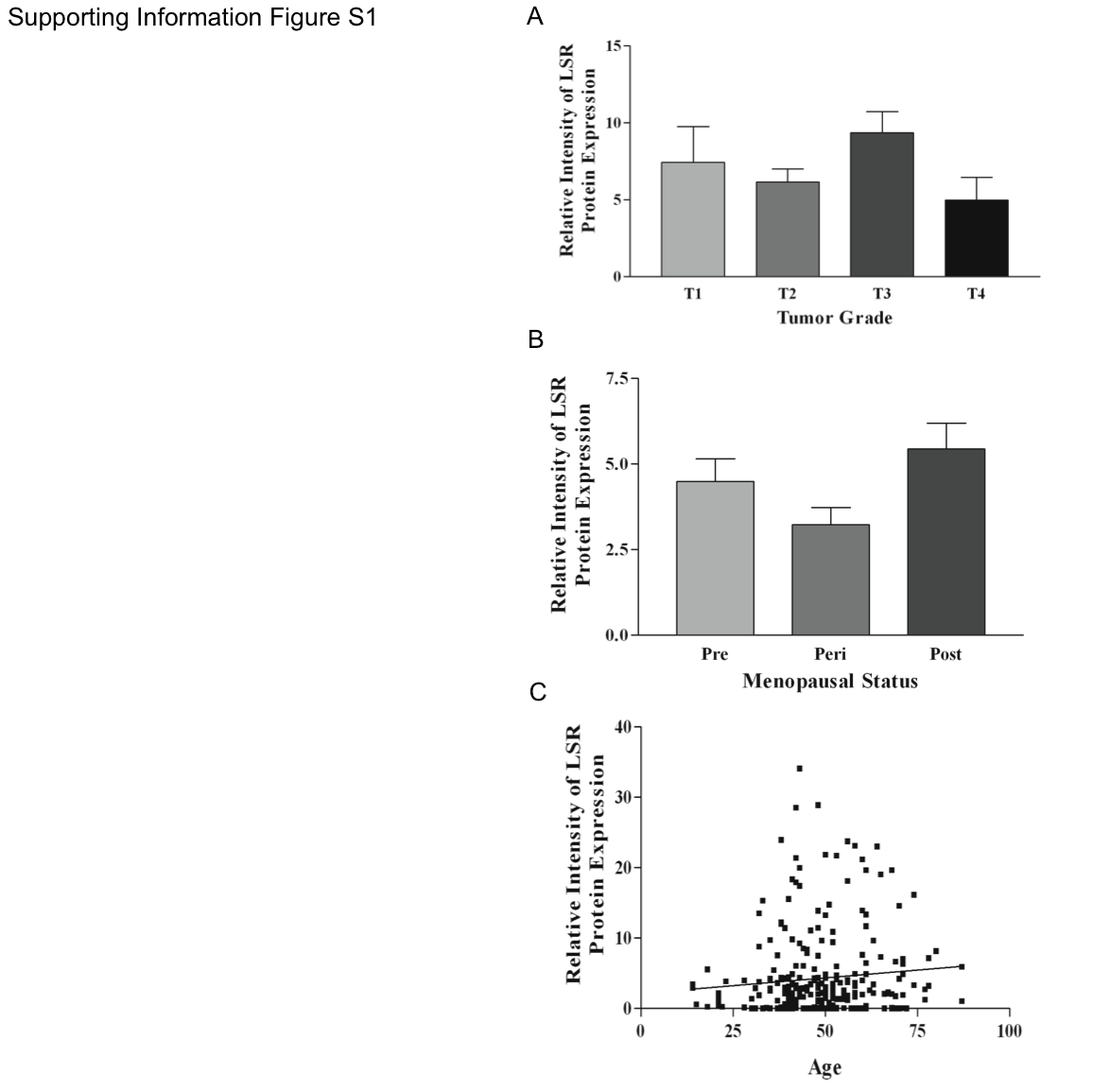

Supplement: Figure S1 — LSR protein expression in breast biopsies and correlation with clinical variables. Breast cancer biopsy tissue arrays were subjected to immunohistochemical analysis using a LSR specific antibody or corresponding negative control. Intensity of LSR expression in correlation with (A) tumor grade, (B) patient menopausal status (premenopausal age 18 to 44 peri-menopausal range 45–53; [41]), and (C) age. Data represent mean relative intensity +/− SE. *P<0.05, **P<0.01. A total of 248 patient samples were analyzed. (TIF) [file pone.0091747.s001.tif]

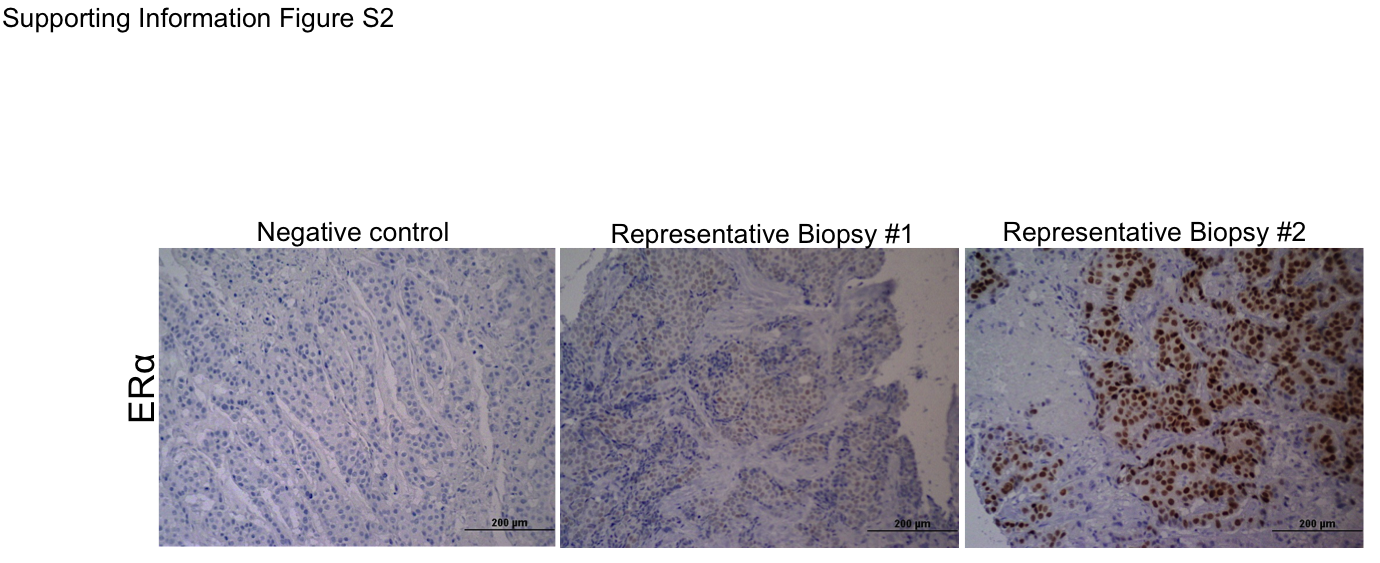

Supplement: Figure S2 — ERα protein expression in breast biopsies. Breast cancer biopsy tissue arrays were subjected to immunohistochemical analysis using an ERα specific antibody or corresponding negative control. Scale bar = 200 uM. A total of 248 patient samples were analyzed. (TIF) [file pone.0091747.s002.tif]

Supporting Information Figure S3

**A**

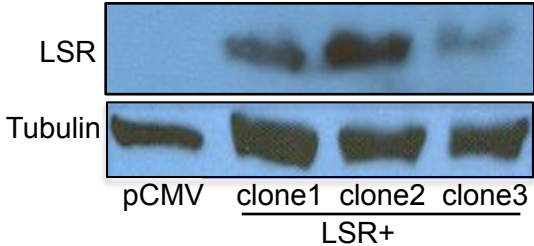

**B**

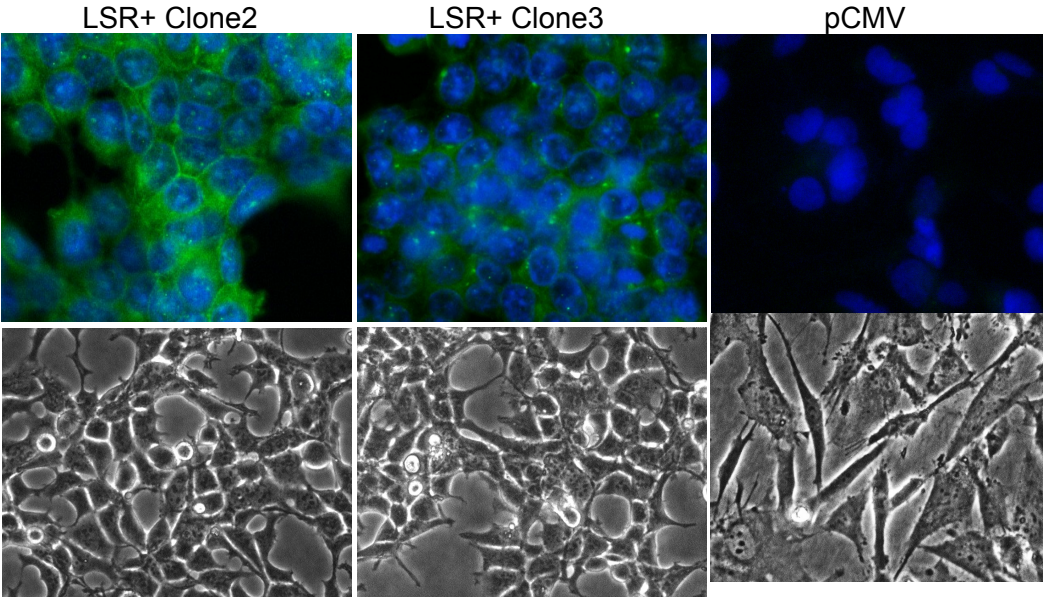

**C**

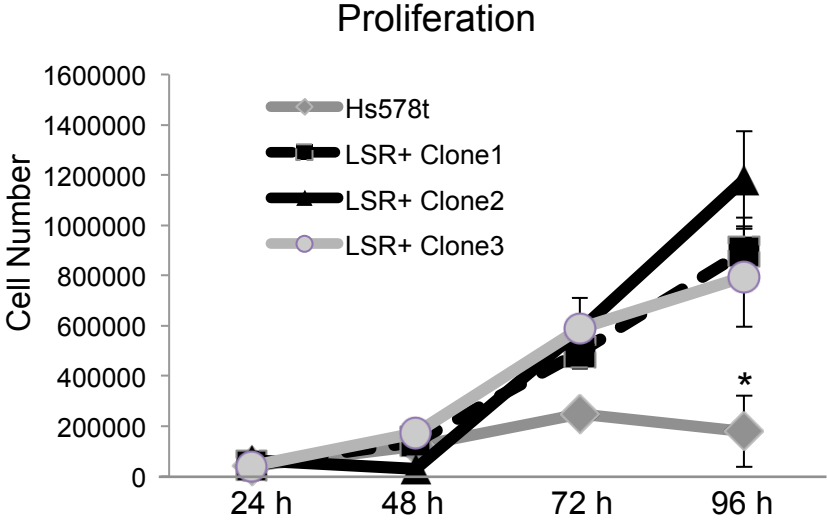

**D**

Tumorsphere Formation

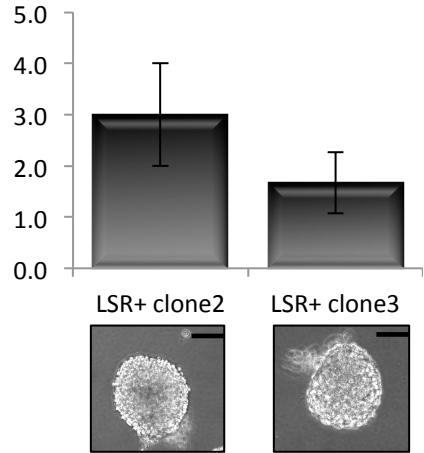

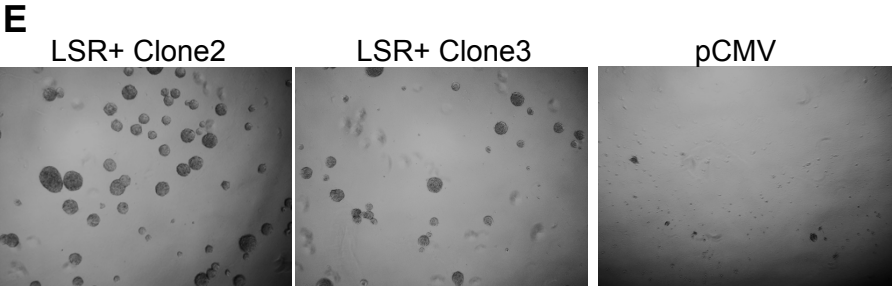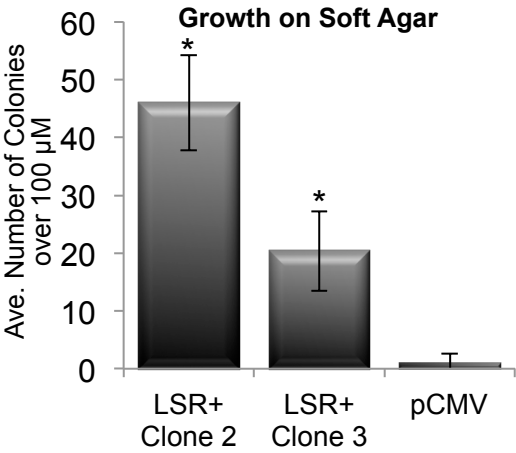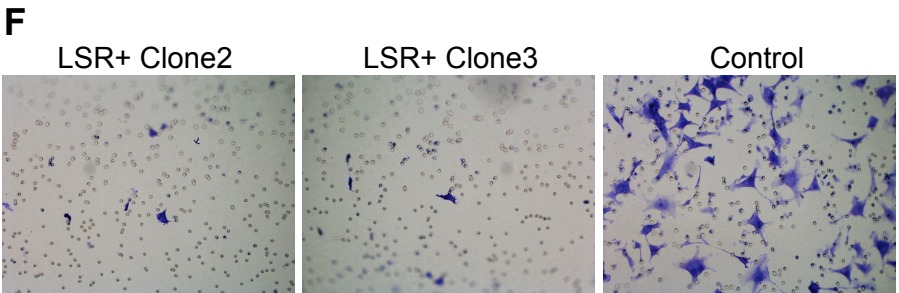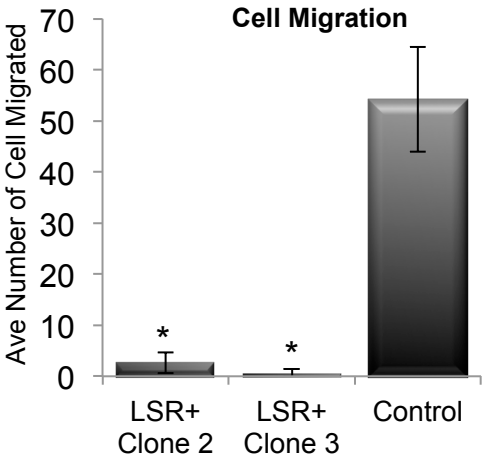

Supplement: Figure S3 — LSR+ Clonal Cell Lines and Functional Assays. Hs578t cells were stably transfected with either a control plasmid (pCMV), or a plasmid containing the full-length gene for LSR variant 1 (LSR+). Clonal cell lines were generated via a single cell plated per well and expanded using the assistance of conditioned media from the parental cell line, in addition to standard culture conditions. (A) Western analysis confirmation of LSR expression. (B) Representative images of immunocytofluorescence using a LSR specific antibody (DNA stained with DAPI). (C) Proliferation assays: cells were plated at 50,000 cells per well in triplicate and counted every 24 h for 96 h. Data represent mean +/− SD. *P<0.01. (D) Sphere forming efficiency: cells were plated in DMEM +10 ng/ml EGF +20 ng/ml FGF +2% B27 in ultralow attachment dishes for seven days then spheres counted and imaged (scale bar, 50 um). (E) Soft agar assays: cells were plated on soft agar coated wells, grown for seven days, and then stained with nitrobluetetrazolium before counting. The entire dish was analyzed and colonies larger than 50 um in diameter were counted. Data represent mean colonies counted per well ± SD; *P<0.001. Top panels are representative images at 20X. (F) Cell migration: Cell were allowed to migrate through transwell inserts towards media containing 10% serum for up to 16 h then fixed, stained and counted (top; representative image of migrated cells, bottom; quantitation). Data represent mean number of cells counted per field ± SD; *P<0.001. (PDF) [file pone.0091747.s003.pdf]
